# USP15-modified ADMSCs-Exo alleviates chondrocyte damage and effectively relieved osteoarthritis by inducing M2 polarization of macrophages through deubiquitinating FOXC1

**DOI:** 10.1186/s13018-025-05742-y

**Published:** 2025-04-02

**Authors:** Qibin Liang, Qinghe Ding, Liang Zhao, Jingchao Tan, Wei Niu

**Affiliations:** 1 The Second Affiliated Hospital of Guangzhou University of Chinese Medicine, Guangzhou University of Chinese Medicine, Guangzhou, 510006 Guangdong China; 2 Department of Orthopaedics, Guangzhou Orthopedic Hospital, Guangzhou, Guangdong 510000 China; 3 Department of Orthopaedics, The Second Affiliated Hospital of Guangzhou University of Chinese Medicine, No. 111, Dade Road, Yuexiu District, Guangzhou, Guangdong Province 510120 China

**Keywords:** OA, USP15, ADMSCs-Exo, Polarization of macrophages, FOXC1, Deubiquitination

## Abstract

**Background:**

The damage to chondrocytes and inflammatory responses are considered the key factors in the pathogenesis of osteoarthritis (OA). Ubiquitin-specific protease 15 (USP15) has been shown to be involved in OA. This study aimed to explore the mechanism of USP15-modified adipose-derived mesenchymal stem cells (ADMSCs) exosome (Exo) in alleviating OA.

**Methods:**

ADMSC-Exo with USP15 overexpression was isolated by magnetic beads method, and the Exo marker proteins were identified by western blot assay. M1 and M2 phenotypic markers of THP1-M0 cells were analyzed by flow cytometry. ELISA was used to detect the expression of inflammatory factors in cells. CCK-8, EdU, Transwell, and flow cytometry were used to detect the cell activity, proliferation, apoptosis and migration ability. The interaction between forkhead box C1 (FOXC1) and USP15 was verified by Glutathione-S-transferase (GST) pull-down and Co-immunoprecipitation (Co-IP) experiments. The stability of FOXC1 was measured by cycloheximide (CHX), and its ubiquitination level was analyzed by exogenous ubiquitination assay.

**Results:**

The Exos from ADMSCs overexpressing USP15 (oe-USP15/Exos) were successfully isolated. It was confirmed that oe-USP15/Exo inhibited the M1 polarization of THP1-M0 cells caused by lipopolysaccharide (LPS) but induced the M2 polarization and the release of inflammatory inhibitory factors. Meanwhile, the damage of chondrocytes caused by LPS was also prevented by oe-USP15/Exo. Besides, USP15 was validated to exert a deubiquitination effect by binding to FOXC1 and positively regulate FOXC1 expression. And the effects of oe-USP15/Exo were abolished after FOXC1 silencing.

**Conclusion:**

USP15-modified ADMSC-derived Exos facilitated M2 polarization of macrophages and improved chondrocyte injury by deubiquitination of FOXC1.

## Introduction

Osteoarthritis (OA), as a degenerative joint disease, has an extremely high incidence rate among middle-aged and elderly populations, primarily manifesting as joint pain and dysfunction [1]. Its pathological mechanisms are complex, involving the gradual degeneration and wear of articular cartilage, as well as accompanying inflammatory responses [2, 3]. Currently, treatment strategies for OA typically involve the use of local or systemic analgesic medications, intra-articular injection of glucocorticoids, and for patients with severe disease, joint replacement surgery may be required for treatment [4, 5]. But these treatment options can only alleviate symptoms and are difficult to fundamentally reverse the disease. Therefore, exploring new therapeutic strategies, especially methods targeting the protection and regeneration of articular cartilage as well as the regulation of inflammation, holds significant clinical importance [6].

In recent years, stem cell therapy has received much attention due to its potential to impel tissue repair and regeneration [7–9]. Compared to other types of mesenchymal stem cells (MSCs), adipose-derived mesenchymal stem cells (ADMSCs) have advantages such as strong proliferation ability, convenient sourcing, multilineage differentiation potential, and immunoregulatory functions, making them one of the preferred types of MSCs for the treatment of various diseases [10–12]. However, direct stem cell transplantation poses numerous risks, such as low cell survival rates and potential immune rejection reactions. Therefore, the use of exosomes (Exos) secreted by stem cells for “acellular” therapy has become a new research hotspot. Exos are lipid bilayer vesicles with a size ranging from 30 to 150 nm, secreted by cells and containing a variety of bioactive substances that are identical to those of the originating cells [13]. They are capable of participating in processes such as intercellular information exchange, immune response, angiogenesis, and tissue repair and regeneration. Previous studies have shown that Exos secreted by ADMSCs can effectively reduce the levels of serum BNP and ANP in rat models of heart failure, while also protecting cardiomyocytes from death, thereby significantly inhibiting the progression of heart failure [14]. ADMSC-Exos have also been proven to effectively replenish mitochondrial components, enhance the mitochondrial integrity and oxidative phosphorylation function of macrophages, thereby facilitating the restoration of metabolism and immune homeostasis in airway macrophages, and alleviating acute lung injury [15]. However, many components in natural MSC-Exo have unclear functions and are susceptible to interference from their source cells and the surrounding microenvironment, which limits the therapeutic effects of Exos and may cause some unexpected side effects. Therefore, it is particularly important to modify Exos using various strategies.

Ubiquitin-specific protease 15 (USP15), as a deubiquitinating enzyme, affects protein degradation and localization by removing ubiquitin molecules, playing a crucial role in various biological processes including cell cycle regulation, signal transduction, and inflammatory responses [16, 17]. It is noteworthy that we have reviewed the literature and found that USP15 inhibits the progression of OA by stimulating TGF-β/SMAD2 signaling and chondrocyte phenotype [18]. Meanwhile, studies have confirmed that USP15 can inhibit the NF-κB pathway, thereby suppressing the pro-inflammatory phenotype of macrophages. Therefore, we hypothesized that USP15-modified ADMSCs-Exo might participate in the development of OA by affecting the polarized phenotype of macrophages.

The objective of this research is to investigate the precise mechanism of USP15-modified ADMSCs-Exo in alleviating OA, hoping to offer new ideas and methods for the treatment of OA, and bring better treatment effect and quality of life for patients.

## Materials and methods

### Cells culture and transfection

This study was approved by the Second Affiliated Hospital of Guangzhou University of Chinese Medicine Ethics Committee and conducted with informed consent from all volunteers. Adipose tissue was obtained from healthy donors and human ADMSCs were isolated from it. Adipose tissues were first washed with PBS buffer and digested with 0.1% collagenase type I (Sigma-Aldrich, St. Louis, MO, USA) and cultured in DMEM (high glucose) medium (Weike Biotechnology, Shanghai, China) containing 10% fetal bovine serum (FBS) (Gibco, Grand Island, NY, USA) and 1% penicillin/streptomycin (P/S) (Invitrogen, Carlsbad, CA, USA), and the medium was replaced with fresh one in a timely manner. Once the cells exhibited the spindle-shaped morphology characteristic, they were subcultured and the fifth-generation ADMSCs were selected for exosome isolation. The THP1-M0, C28/I2, and HEK-293T cells were obtained from Cancer Institute of the Chinese Academy of Medical Sciences (Beijing, China). Among them, the THP1-M0 cells were suspension cells and required RPMI-1640 medium (Invitrogen) supplemented with 10% FBS (Gibco) and 1% P/S (Invitrogen); whereas the C28/I2 and HEK-293T cells were adherent cells that required culturing in high-glucose DMEM medium (Weike Biotechnology) with 10% FBS (Gibco) and 1% P/S (Invitrogen).

Using the empty vector pcDNA3.1 (Invitrogen) (oe-NC) as the negative control group, the pcDNA3.1-USP15 (oe-USP15) were transiently transfected into ADMSCs cells by Lipofectamine 2000 reagent (Invitrogen), which was to achieve overexpression of USP15 in these cells. Additionally, the sh- forkhead box C1 (FOXC1) was purchased from GeneChem (Shanghai, China) and transiently transfected into the THP1-M0 and C28/I2 cells using Lipofectamine 2000 reagent (Invitrogen) to knock down the expression of FOXC1.

### Identification of ADMSCs

ADMSCs were passaged to the fifth generation, followed by the use of flow cytometry to detect mesenchymal markers CD90 (ab288825, Abcam, Cambridge, UK) and CD44 (ab264539, Abcam), as well as hematopoietic cell surface markers CD45 (ab243869, Abcam) and CD34 (ab315802, Abcam). To evaluate the multilineage differentiation potential of ADMSCs, their differentiation were induced using adipogenic induction medium and chondrogenic induction medium, and the differentiation effects of ADMSCs were observed through Oil Red O (CAS#: 1320-06-5, Sigma) staining and Alcian Blue (CAS#: 33864-99-2, Sigma) staining.

### Isolation and characterization of exos

When the density reached about 80%, ADMSCs were washed with PBS and then continued to be cultured in serum-free stem cell medium for 48 h. The supernatant was collected and first centrifuged at 300×g for 10 min, then centrifuged again at 2,000×g for 10 min using a centrifuge at 4 ℃. The supernatant was collected once more and filtered through a 0.22 μm filter to remove cell debris. The Amicon Ultra-15 filter was used to centrifuge the sample at 4,000×g and 4 ℃ until the volume was reduced to 200 µL. After washing with PBS, it was ultrafiltered again to 200 µL. The Exos were then coated on a 30% sucrose /D_2_O mat in an Ultra-Clear tube by centrifugation at 100,000×g for 1 h at 4 ℃ and partially purified Exos were recovered using an 18 g needle. The PBS dilution was then passed through a filter device at 4 ℃ and centrifuged at 4,000 g to a volume of 200 µL. The purified ADMSCs-derived Exos could be used immediately or maintained at -80℃. Transmission electron microscopy (TEM) was used to observe the morphology of exosomes, and Nanoparticle Tracking Analysis (NTA) was employed to analyze the particle size and concentration of Exos. Finally, the expression of Exo marker proteins was identified by western blot (WB) using antibodies against TSG101 (ab125011, Abcam), CD81 (ab79559, Abcam) and CD9 (ab236630, Abcam). The supernatant was collected as described above to obtain Exos. The Exos secreted by cells transfected with empty overexpression vector were divided into oe-NC/Exo group, and Exos secreted by cells transfected with USP15 overexpression plasmid were divided into oe-USP15/Exo group.

### Co-culture of oe-USP15/Exo with THP1-M0, C28/I2 cells

THP1-M0 and C28/I2 cells were co-cultured with lipopolysaccharide (LPS, Sigma) (50 ng/mL) or LPS + 1 × 10^11^ ADMSCs-derived Exos for 24 h. Cells or supernatants were then respectively collected for subsequent experiments.

### Uptake of exos

Firstly, the DiI dye solution (Beyotime, Shanghai, China) was diluted with PBS and incubated with Exos. After centrifugation to remove excess dye, the precipitate was washed with PBS. The DiI-labeled Exos were then co-cultured with THP1-M0 and C28/I2 cells for 24 h using the method described above. After washing with PBS, the cells were fixed with 4% paraformaldehyde (PFA). Finally, the uptake of DiI-labeled Exos was observed using a laser scanning confocal microscope after staining the cell nuclei with DAPI.

### Western blot (WB) assay

The cells were lysed with RIPA lysate (Beyotime) containing protease inhibitors and centrifuged to remove cell debris. The protein concentration was detected by the BCA Protein Assay Kit (Beyotime) and then subjected to SDS-PAGE. At the end of electrophoresis, the plates were blotted onto PVDF membranes (Millipore, Billerica, MA, USA) and incubated overnight with primary antibodies USP15 (ab71713, Abcam) and FOXC1 (ab227977, Abcam), followed by continued incubation with the corresponding species-specific secondary antibody Goat Anti-Rabbit IgG H&L (HRP)(ab6721, Abcam). Finally, the results were detected and imaged by RapidStep ECL Reagent (Millipore Corp., Billerica, MA, United States) with GAPDH gene as the internal reference gene.

### Real-time quantitative PCR (RT-qPCR)

Total RNA was extracted using Trizol reagent (Invitrogen) and mRNA reverse transcribed using the Transcriptor First Strand cDNA Synthesis Kit (Roche, Vilvoord, Brussel, Belgium) to generate total cDNA. The quantitative PCR reactions were performed on C1000 thermal cycler (Bio-Rad Laboratories Inc., Hercules, CA, USA) with SYBR Premix Ex Taq II (TaKaRa, Dalian, China) using cDNA as a template. The primer sequences were shown in Table 1.

**Table 1:** Primer sequences for RT-qPCR

Name		Primers for PCR (5’ -3’)
USP15	Forward	CGACGCTGCTCAAAACCTC
	Reverse	TCCCATCTGGTATTTGTCCCAA
GAPDH	Forward	ACAACTTTGGTATCGTGGAAGG
	Reverse	GCCATCACGCCACAGTTTC

### Flow cytometry

Using the Annexin V-FITC/PI Apoptosis Detection Kit (Yeasen, Shanghai, China), the effect of USP15-overexpressing Exos on the apoptosis of LPS-treated C28/I2 cells were evaluated by flow cytometry. The specific steps were as follows: First, the cells were resuspended in 1×Binding buffer. Then, Annexin V (10 µL) and propidium iodide (PI) (5 µL) reagents were added to stain for 15 min in the dark. After staining, the cells were washed with PBS and resuspended in 1×Binding buffer. Finally, flow cytometry analysis was performed with FITC as the x-axis and PI (PE-A) as the y-axis.

To investigate the specific role of USP15-overexpressing Exos on LPS-induced polarization of THP1-M0 cells, the following experimental procedures were conducted: Firstly, the USP15-overexpressing Exos were co-cultured with LPS-treated THP1-M0 cells to induce their polarization. Subsequently, the cells were collected and fixed with 4% paraformaldehyde for 20 min and washed with permeabilization buffer. Under light-shielded conditions at 4 ℃, we stained the cells with antibodies against iNOS (ab283655, Abcam), a marker of the M1 phenotype of macrophages, and CD206 (ab270647, Abcam), a marker of the M2 phenotype, respectively, and washed them again. Finally, the expression of iNOS and CD206 were detected by flow cytometry analysis.

### Enzyme-linked immunosorbent assay (ELISA)

The levels of cytokines in the supernatant of THP1-M0 cells were detected by ELISA assay. The human IL-1β, TNF-α, IL-6, IL-10, and TGF-β ELISA kits purchased from Yeasen were utilized to measure the release of the above inflammatory factors.

### Cell viability assay

The effect of oe-USP15/Exo on the viability of LPS-treated C28/I2 cells was verified using the Cell Counting Kit-8 (Beyotime). The C28/I2 cells were seeded into 96-well plates and cultured until adherent. After the cells were treated according to different experimental groups for 24 h, 10 µL of CCK-8 solution was added to each well and incubated for 2 h. Cell viability was analyzed by measuring the absorbance at 450 nm using a microplate reader.

### 5-Ethynyl-2’-deoxyuridine (EdU) assay

The Yefluor488 EdU Cell Imaging Kit (Yeasen) was used to observe the proliferation of cells. After LPS and oe-USP15/Exo treatment, C28/I2 cells were incubated with EdU working solution in an incubator for 3 h. Subsequently, the medium was removed and 4% paraformaldehyde was added for incubation at room temperature for 20 min. Then, glycine solution was added for 5 min to neutralize the residual fixative, followed by two washes with PBS wash solution. Next, 0.5% Triton X-100 was added to facilitate permeabilization for 20 min. The Click-iT reaction mixture was prepared and added to each well. The culture plate was gently shaken to ensure uniform coverage of the cells with the reaction mixture. After washing by PBS buffer, 1×Hoechest 33,342 (5 µg/mL) was added to stain the cells nuclei in the dark. Finally, the effect of oe-USP15/Exo on the proliferation of LPS-treated C28/I2 cells was observed under a fluorescence microscope.

### Transwell assay

The migration characteristics of C28/I2 cells were evaluated using the Transwell chamber assay (Corning, Tewksbury, MA, USA). During the experiment, cells were uniformly seeded in the upper chamber without serum and incubated overnight. The next day, cells that had migrated to the lower chamber containing serum were fixed with methanol, stained with 0.1% crystal violet for 10 min, and washed with PBS buffer. Finally, the cells were counted under a microscope.

### Glutathione-S-transferase (GST) pull-down

The HEK-293T cells were harvested and washed with precooled PBS, then centrifuged after adding cell lysate containing protease inhibitors, the supernatant was collected and a portion of the supernatant was used as input samples. The decoy expression vectors of FOXC1 and USP15 tagged with GST were transformed into *E. coli* BL21. After expansion, the cells were collected and washed, and the lysates and protease inhibitors were added. The bait protein lysate was added to the prepared magnetic bead suspension, incubated and allowed to stand, and after clarification, the supernatant was discarded and washed several times to remove unbound proteins. The prepared capture protein lysate was then added to continue the incubation, and the magnetic beads were washed again. Then the binding proteins were eluted from the beads and denatured by boiling. The expression of USP15 and FOXC1 in the eluted protein samples was detected by WB to verify the interaction between them.

### Co-immunoprecipitation (Co-IP) assay

The Co-IP assay was utilized to demonstrate the interaction between USP15 and FOXC1 in THP1-M0 and C28/I2 cells. The cells were respectively collected, after centrifugation, the supernatant was removed and precooled cell lysate was added. The lysed cell suspension was centrifuged to remove cell debris. The collected supernatants were then divided into three groups, Input group, control antibody IgG added to one group, and USP15 or FOXC1 antibody added to the other group, and incubated overnight at 4 ℃. Subsequently, following the antibody incubation, the Protein A/G agarose beads (Yeasen) were introduced into the supernatant and allowed to co-incubate at a temperature of 4 ℃ for 2 h, in order to effectively capture the protein complexes, followed by centrifugation and washing of the beads with precooled wash buffer. The loading buffer was added to the washed agarose beads and boiled for WB assay.

### Protein stability assay

Cicloheximide (CHX), also known as cycloheximide, is an organic compound that inhibits protein synthesis. To disrupt the synthesis of FOXC1, the cells were treated with CHX. Specifically, we first overexpressed USP15 in THP1-M0 cells and C28/I2 cells, and then treated these cells with CHX. Protein samples were collected at 0, 2, 4, and 8 h after treatment, and western blot assay was used to verify the effect of USP15 overexpression on the FOXC1 level.

### Determination of protein ubiquitination levels

After successfully overexpressing USP15 in HEK-293T cells, the cells were lysed with cell lysis buffer containing protease inhibitors and obtained the cell supernatant through centrifugation. To stabilize protein-protein interactions, dimethyl sulfoxide (DMSO) was added to the cell supernatant. Next, ubiquitin antibody or FOXC1 antibody was bound to Protein A/G agarose beads, added the lysed cell supernatant, and incubated the mixture on a shaker. After washing, the samples were boiled for subsequent WB experiments. The aim of this experiment was to detect the ubiquitination level of FOXC1 under the condition of overexpressing USP15.

### Statistical analysis

All experiments were repeated three times to ensure the reliability of the results. Statistical analysis was performed using Graphpad Prism 7 software, and the comparison between two groups or among multiple groups was conducted using the Student’s *t*-test or one-way analysis of variance (ANOVA) method. All data obtained were reported as mean ± standard deviation. Unless stated otherwise, a *P*-value < 0.05 was deemed significant.

## Results

### Isolation and identification of ADMSCs-Exo overexpressing USP15

**Fig. 1:**
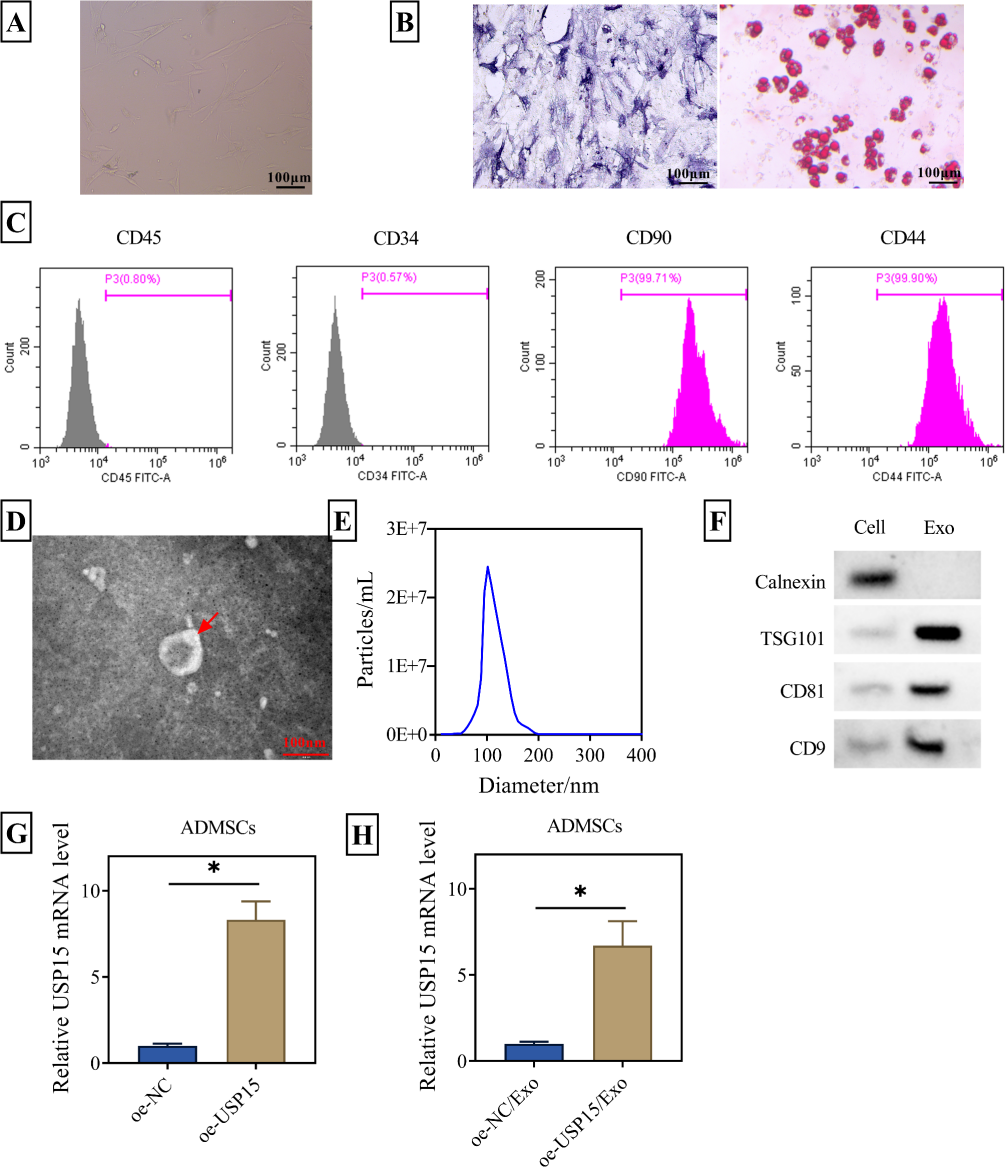
Isolation and identification of oe-USP15/Exo. (**A**) Morphology of ADMSC. Scale bar: 100 μm. (**B**) The chondrogenic and adipogenic differentiation ability of ADMSC was determined by Alician blue and Oil Red O staining kits. Scale bar: 100 μm. (**C**) Flow cytometry analysis of characteristic cell surface markers of ADMSCs. The hematopoietic markers CD45 and CD34 were negative, while the mesenchymal markers CD90 and CD44 were positive. (**D**) The morphology of Exos was observed by transmission electron microscope. Scale bar: 100 nm. (**E**) NTA for Exo particle size and concentration. (**F**) WB detection of the expression of Exo marker proteins CD9, CD81, TSG101 and negative protein Calnexin. (**G**) RT-qPCR was utilized to detect the mRNA levels of ADMSCs and ADMSCs-Exo after USP15 overexpression. *P < 0.05

The bright-field image showed the morphology of ADMSCs as depicted in Fig. 1A. The staining results with Alcian Blue and oil red O clearly demonstrated the ability of ADMSCs to differentiate into multiple cell types, including chondrocytes and adipocytes (Fig. 1B). With the help of flow cytometry, it was found that the hematopoietic markers CD45 and CD34 were negative, while the mesenchymal markers CD90 and CD44 were positive in ADMSCs (Fig. 1C). Using ultrafiltration and centrifugation techniques, the Exos from the supernatant of ADMSCs were successfully isolated. And under a transmission electron microscope, the Exo derived from ADMSCs exhibited a typical ring-like structure, as shown in Fig. 1D. The average vesicle diameter of ADMSCs-Exo was about 100 nm as determined by nanoparticle tracking analyzer (Fig. 1E). Simultaneously, the expression of Exo marker proteins CD9, CD81, and TSG101 were displayed by WB assay, while the negative control protein Calnexin was not detected (Fig. 1F). Then USP15 was overexpressed in ADMSCs. And the high expression of USP15 in ADMSCs as well as ADMSCs-Exos was also determined (Fig. 1G-H). These results confirmed our successful isolation of ADMSCs-derived Exos overexpressing USP15.

### ADMSCs-Exo overexpressing USP15 shifted the polarization of macrophages from M1 to M2 phenotype

**Fig. 2:**
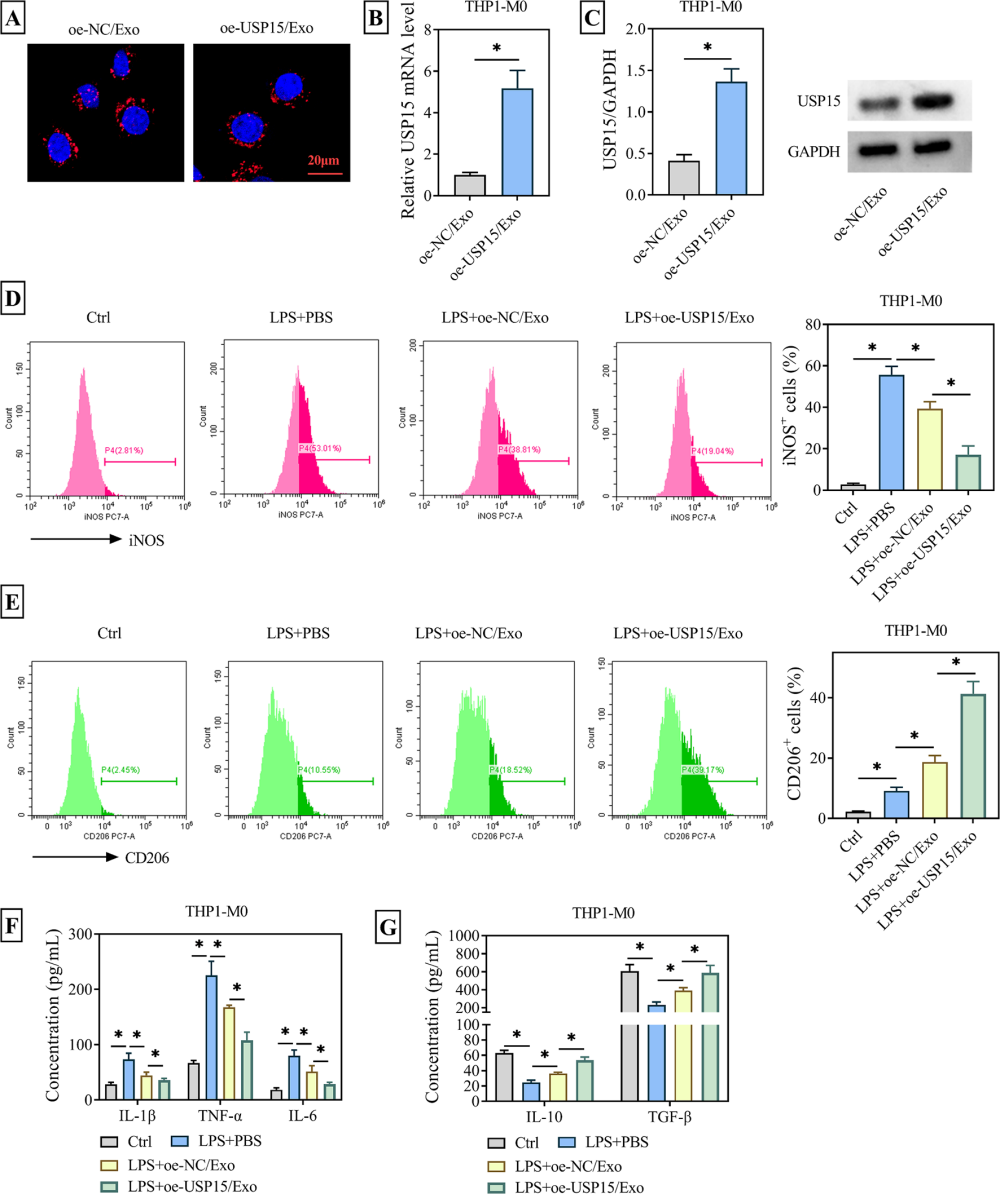
Effects of ADMSCs-Exo overexpressing USP15 on macrophage polarization. Cells were randomly divided into four groups: Ctrl group (no treatment); LPS + PBS group (complete medium containing 10 µg/mL LPS + PBS buffer); LPS + oe-NC/Exo group (complete medium containing 10 µg/mL LPS + ADMSCs/Exo transfected with pcDNA3.1); LPS + oe-USP15/Exo group (complete medium containing 10 µg/mL LPS + ADMSCs/Exo transfected with pcDNA3.1-USP15). (**A**) DiI-labeled ADMSCs-Exo with USP15 overexpression was co-incubated with THP1-M0 cells to detect Exo uptake. (**B**-**C**) RT-qPCR and WB assays were used to detect the expression of USP15 in THP1-M0 cells after co-incubation of THP1-M0 with USP15-overexpressing ADMSCs-Exo. (**D**) The expression of M1 phenotypic marker iNOS in LPS-treated THP1-M0 cells was detected by flow cytometry. (**E**) The expression of M2 phenotypic marker CD206 was detected by flow cytometry. (**F**) ELISA was used to detect the expression of pro-inflammatory cytokines IL-6, IL-1β, TNF-α and anti-inflammatory cytokines IL-10, TGF-β in THP1 cells. *P < 0.05

To examine whether isolated ADMSC Exos overexpressing USP15 had an impact on macrophages polarization, oe-USP15/Exos were co-cultured with THP1-M0 cells. The Exos were labeled with DiI, while the cell nuclei were stained with DAPI. Observation under a laser scanning confocal microscope confirmed the successful internalization of oe-USP15/Exo into THP1 cells (Fig. 2A). At the same time, WB and RT-qPCR assays verified that USP15 was highly expressed at both mRNA and protein levels in THP1 cells co-cultured with oe-USP15/Exo (Fig. 2B-C). Known as an endotoxin derived from bacteria, LPS is characterized by its ability to trigger the release of proinflammatory cytokines, thereby inducing and initiating an inflammatory response [19]. Thus, LPS treatment was employed to mimic the symptoms of OA. Flow cytometry showed that compared with the Ctrl group, the expression of M1 phenotype marker iNOS was greatly increased, while the expression of M2 phenotype marker CD206 was also slightly increased in the LPS/PBS treatment group. Meanwhile, treatment of THP1 cells with LPS/oe-NC/Exo significantly curbed the expression of iNOS and elevated the expression of CD206. Moreover, treatment of LPS-treated THP1 cells with Exos secreted by ADMSCs overexpressing USP15 resulted in a more significant decrease in iNOS and an even greater increase in CD206 (Fig. 2D-E). In addition, ELISA results indicated that under the above treatment conditions, the release of pro-inflammatory cytokines IL-1β, TNF-α and IL-6 in THP1 cells of LPS + PBS group was drastically increased, while the levels of anti-inflammatory cytokines IL-10 and TGF-β were decreased. However, in the presence of ADMSCs-secreted Exos, the release of IL-1β, TNF-α and IL-6 was obviously hindered, while the release of IL-10 and TGF-β was restored. More importantly, Exos derived from ADMSCs with USP15 overexpression had a more significant effect on the release of inflammatory factors in THP1 cells than oe-NC/Exos (Fig. 2F-G). In conclusion, Exos derived from ADMSC overexpressing USP15 induced the polarization of macrophage from M1 to M2 phenotype and increased the release of anti-inflammatory factors.

### ADMSCs-Exo overexpressing USP15 alleviated LPS-induced chondrocyte damage

**Fig. 3:**
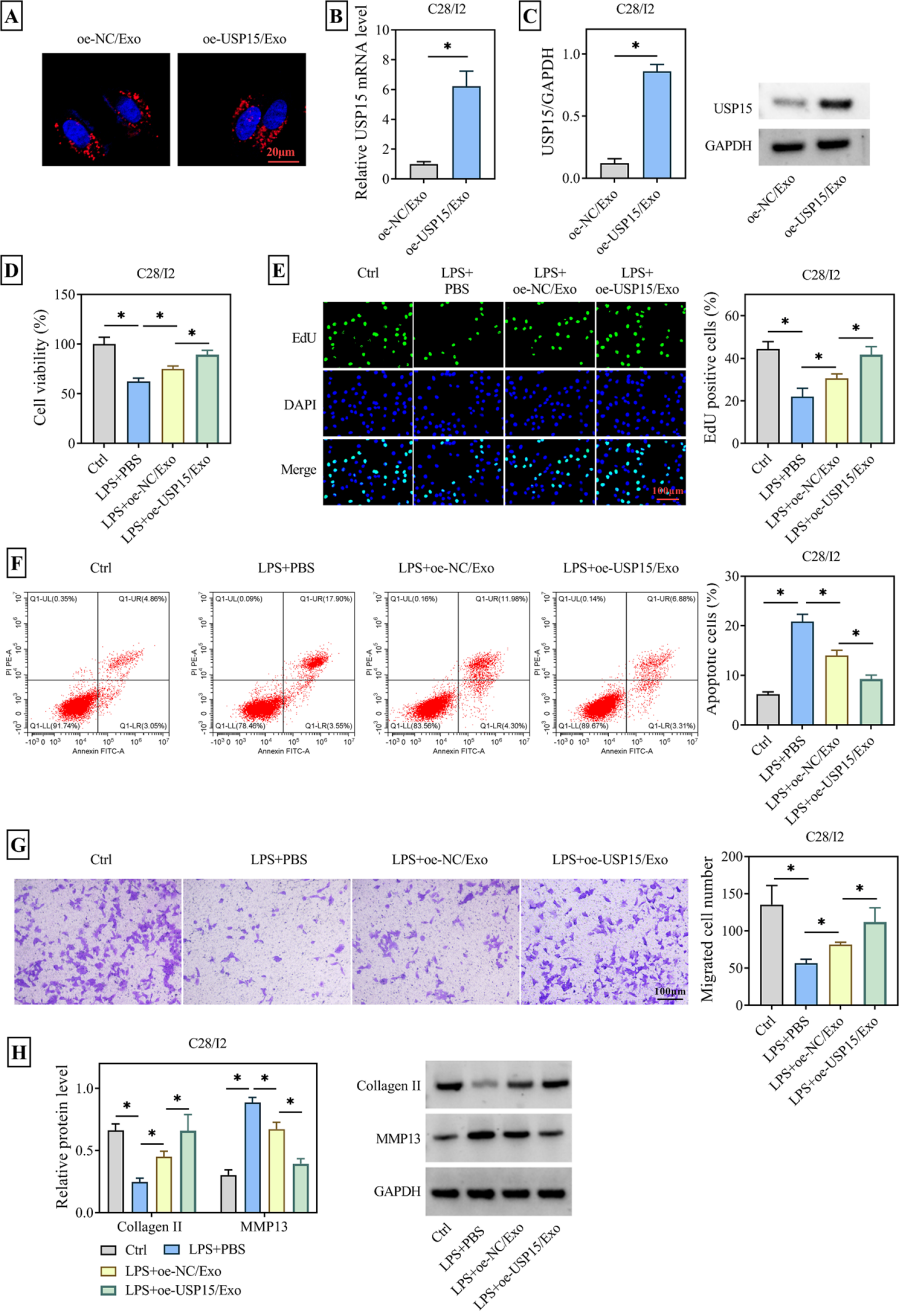
Effects of oe-USP15/Exo on LPS-induced chondrocyte injury. Cells were randomly divided into four groups: Ctrl group; LPS + PBS group; LPS + oe-NC/Exo group; LPS + oe-USP15/Exo group. (**A**) DiI-labeled ADMSCs-Exo with USP15 overexpression was co-incubated with C28/I2 cells to detect Exo uptake. (**B**-**C**) RT-qPCR and WB assays were employed to detect the expression of USP15 in C28/I2 cells after co-incubation of C28/I2 with USP15-overexpressing ADMSCs-Exo. (**D**) The effect of oe-USP15/Exo on the viability of LPS-treated C28/I2 cells was determined by CCK-8 assay. (**E**) EdU assay was used to detect the effect of oe-USP15/Exo on proliferation of LPS-treated C28/I2 cells. (**F**) Using flow cytometry to analyze the effect of oe-USP15/Exo on apoptosis in LPS-treated C28/I2 cells. (**G**) The effect of oe-USP15/Exo on the migration of LPS-treated C28/I2 cells was determined by Transwell assay. (**H**) WB was used to detect the expression of proteins involved in cartilage matrix degradation (Collagen II) and remodeling (MMP13). *P < 0.05

Then, the oe-USP15/Exo we co-cultured with chondrocyte C28/I2 and still used DiI to label the Exos. Fluorescence microscopy was utilized to observe DiI-labeled Exos within the cytoplasm of C28/I2 cells, confirming their uptake (Fig. 3A). And it was also determined that USP15 was highly expressed in C28/I2 cells compared with the control group (Fig. 3B-C). Subsequently, the cells were divided into four treatment groups: Ctrl group, LPS group, LPS + oe-NC/Exo group and LPS + oe-USP15/Exo group. Each group was subjected to corresponding treatments, followed by analysis of some cellular behaviors. CCK-8 and EdU experiments revealed that LPS treatment markedly suppressed the activity and proliferation of C28/I2 cells. However, when these cells were co-cultured with ADMSCs/Exo, their activity and proliferation were notably revitalized. Furthermore, the restorative effect observed with oe-USP15/Exo was even more pronounced (Fig. 3D-E). Flow cytometry results also confirmed that Exos secreted by ADMSCs overexpressing USP15 exhibited a greater degree of inhibition on the apoptosis rate of C28/I2 cells (Fig. 3F). Meanwhile, as shown in Fig. 3G, compared with the LPS + PBS group, the migration ability of C28/I2 cells in the LPS + oe-NC/Exo group and the LPS + oe-USP15/Exo group was increased, and the effect of the LPS + oe-USP15/Exo group was better. Additionally, oe-USP15/Exo upregulated the Collagen II protein, a component closely associated with cartilage matrix degradation that was downregulated by LPS treatment. Conversely, the elevation of MMP3 protein, another factor involved in cartilage matrix degradation induced by LPS, was reversed upon co-culturing C28/I2 cells with oe-USP15/Exo (Fig. 3H). The above results confirmed that oe-USP15/Exo was able to ameliorate LPS-induced chondrocyte injury.

### USP15 interacted with and deubiquitinated FOXC1

**Fig. 4:**
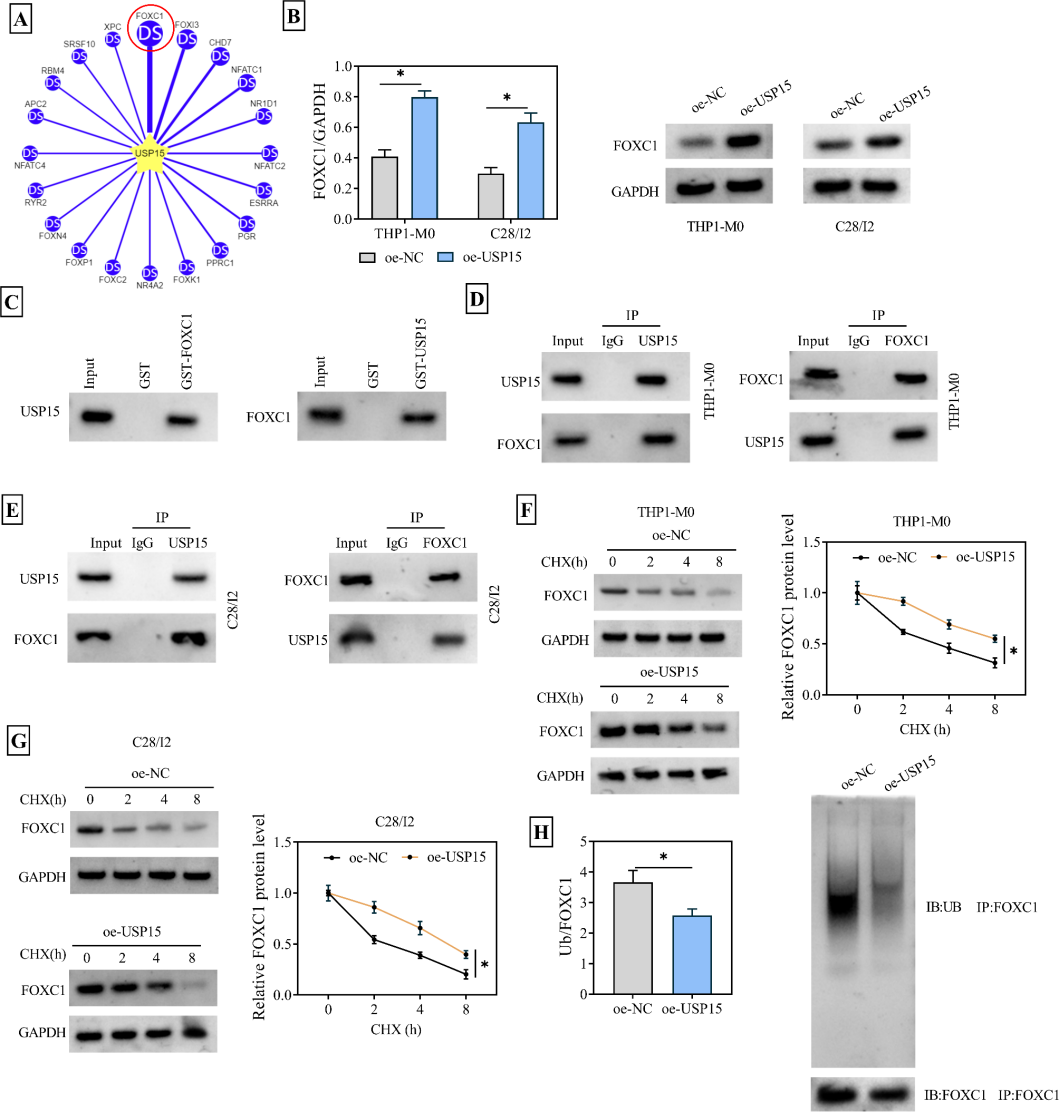
USP15 stabilizes the FOXC1 protein through deubiquitination. THP1-M0 and C28/I2 cells were transfected with pcDNA3.1 (oe-NC) and pcDNA3.1-USP15 (oe-USP15). (**A**) The top 20 target proteins of USP15 were predicted by Ubibrowse_v2. (**B**) WB assay was used to detect the protein levels of FOXC1 in THP1-M0 and C28/I2 cells after USP15 overexpression. (**C**) GST pull-down assay was used to detect the interaction between FOXC1 and USP15 in HEK-293T cells. (**D**) The interaction between USP15 and FOXC1 in THP1-M0 cells was detected using Co-IP assay. (**E**) The interaction between USP15 and FOXC1 in C28/I2 cells was determined by Co-IP assay. (**F**-**G**) WB assay was used to measure the stability of FOXC1 protein in THP1-M0 and C28/I2 cells after CHX treatment. (**H**) The ubiquitination level of FOXC1 in HEK-293T cells overexpressing USP15 was analyzed by exogenous ubiquitination assay. *P < 0.05

The Ubibrowse_v2 website was utilized to screen the top 20 target proteins of USP15 and select the highest-scoring FOXC1 protein for subsequent research efforts (Fig. 4A). The pcDNA3.1-USP15 plasmid was transfected into THP1-M0 and C28/I2 cells, and WB analysis revealed that when USP15 was overexpressed, the level of FOXC1 also showed a corresponding upregulation trend (Fig. 4B). Is there an interaction between USP15 and FOXC1? Therefore, a GST pull-down assay was performed, which clarified that compared with the Input group and the GST control group, the GST-FOXC1 fusion protein were successfully pulled down by the USP15 antibody. Simultaneously, the GST-USP15 fusion protein was also precipitated by the action of the FOXC1 antibody (Fig. 4C). Furthermore, Co-IP assay in THP1-M0 and C28/I2 cells were respectively conducted, confirming the genuine interaction between USP15 and FOXC1 (Fig. 4D-E). It was noteworthy that when THP1-M0 and C28/I2 cells transfected with pcDNA3.1-USP15 were treated with CHX, an interesting phenomenon was observed: although the expression of FOXC1 in the oe-USP15 group of THP1-M0 cells decreased over time, the gray value of the oe-USP15 group was abnormally higher than that of the oe-NC group at the same time point. This result indicated that USP15 had a stabilizing effect on the expression level of FOXC1 (Fig. 4F-G). Given that USP15 was a well-known deubiquitinating enzyme, an exogenous ubiquitination experiment was conducted and found that the ubiquitination level of FOXC1 was exceptionally retarded when USP15 was overexpressed (Fig. 4H). In brief, USP15 positively regulated FOXC1 expression through deubiquitination.

### Exos derived from ADMSC overexpressing USP15 induced macrophage polarization from M1 to M2 by upregulating FOXC1

**Fig. 5:**
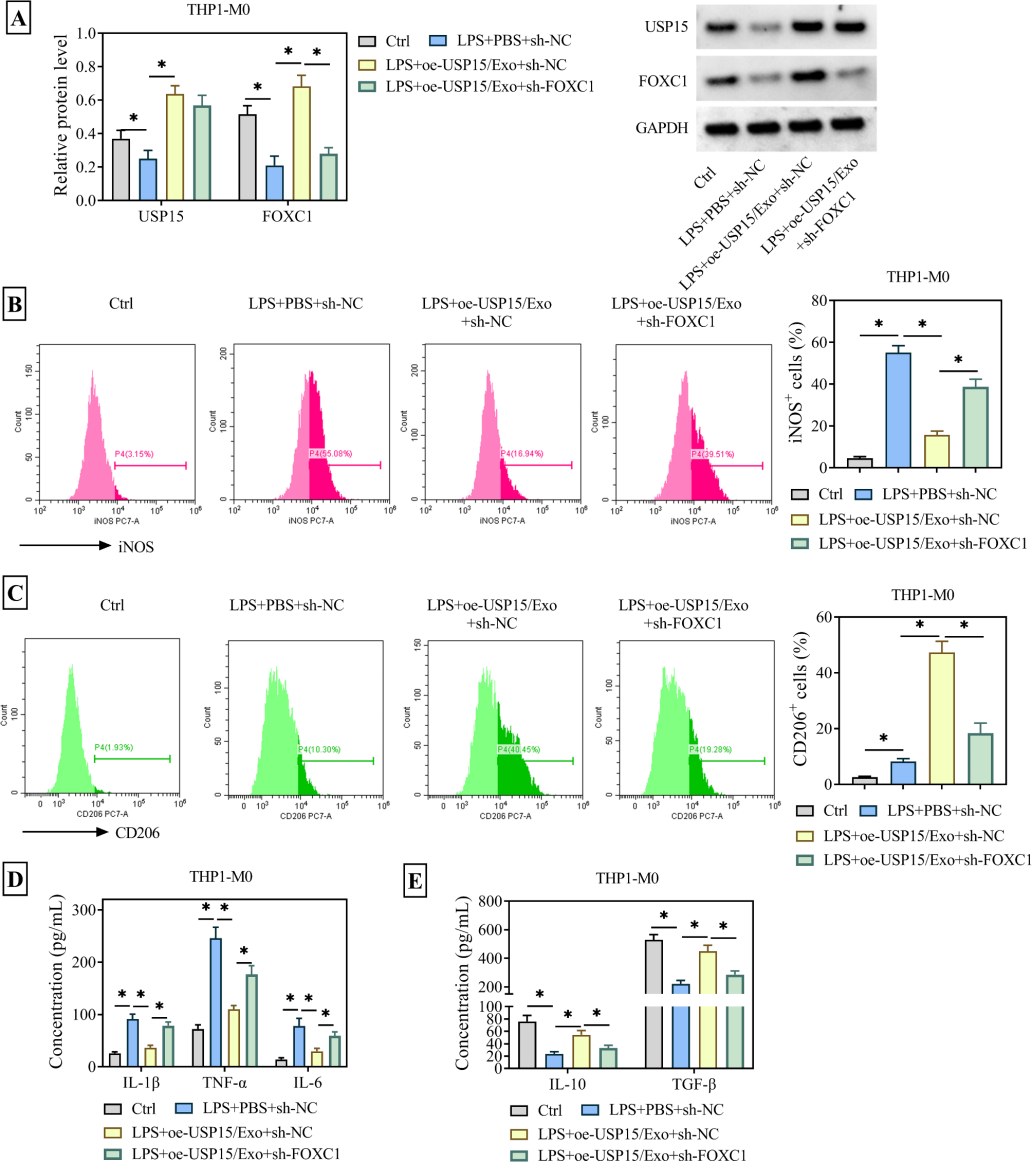
Knockdown of FOXC1 abolishes the effects of USP15-modified Exos on macrophage polarization. THP1-M0 cells were randomly divided into four groups: Ctrl group (no treatment); LPS + PBS + sh-NC group (10 µg/mL LPS + PBS buffer + transfected with sh-NC); LPS + oe-NC/Exo + sh-NC group (10 µg/mL LPS + ADMSCs/Exo transfected with pcDNA3.1 + transfected with sh-NC); LPS + oe-USP15/Exo + sh-FOXC1 group (10 µg/mL LPS + ADMSCs/Exo transfected with pcDNA3.1-USP15 + transfected with sh-FOXC1). (**A**) WB was used to verify the protein expression of USP15 and FOXC1 in LPS-treated THP-1-M0 cells stably transfected with sh-FOXC1 after co-incubation with oe-USP15/Exo. (**B**) The expression of M1 phenotypic marker iNOS in THP1 cells was detected by flow cytometry. (**C**) The expression of M2 phenotypic marker CD206 in THP1 cells was detected by flow cytometry. (**D**) ELISA was used to detect the expression of pro-inflammatory cytokines IL-6, IL-1β, TNF-α and anti-inflammatory cytokines IL-10, TGF-β in THP1 cells. *P < 0.05

So did the USP15 and FOXC1 affect macrophage polarization? The sh-FOXC1 was transfected into LPS-treated THP1-M0 cells co-cultured with oe-USP15/Exo, and the levels of USP15 and FOXC1 were verified by WB. It was found that the expression of USP15 and FOXC1 was inhibited by LPS alone, but was upregulated in the LPS + oe-USP15/Exo group (Fig. 5A). As expected, when FOXC1 was silenced, the expression of iNOS was increased, whereas the expression of CD206 was downregulated (Fig. 5B-C). Of course, when FOXC1 was silenced, the release of the proinflammatory cytokines IL-1β, TNF-α and IL-6 by THP1-M0 cells significantly increased, while at the same time, the release of the anti-inflammatory cytokines IL-10 and TGF-β were evidently suppressed (Fig. 5D-E). In summary, this indicated that ADMSC-Exo overexpressing USP15 induced macrophages polarization from M1 to M2 by facilitating the expression of FOXC1 protein.

### ADMSC-Exo overexpressing USP15 alleviated LPS-induced chondrocyte damage by upregulating FOXC1 protein

**Fig. 6:**
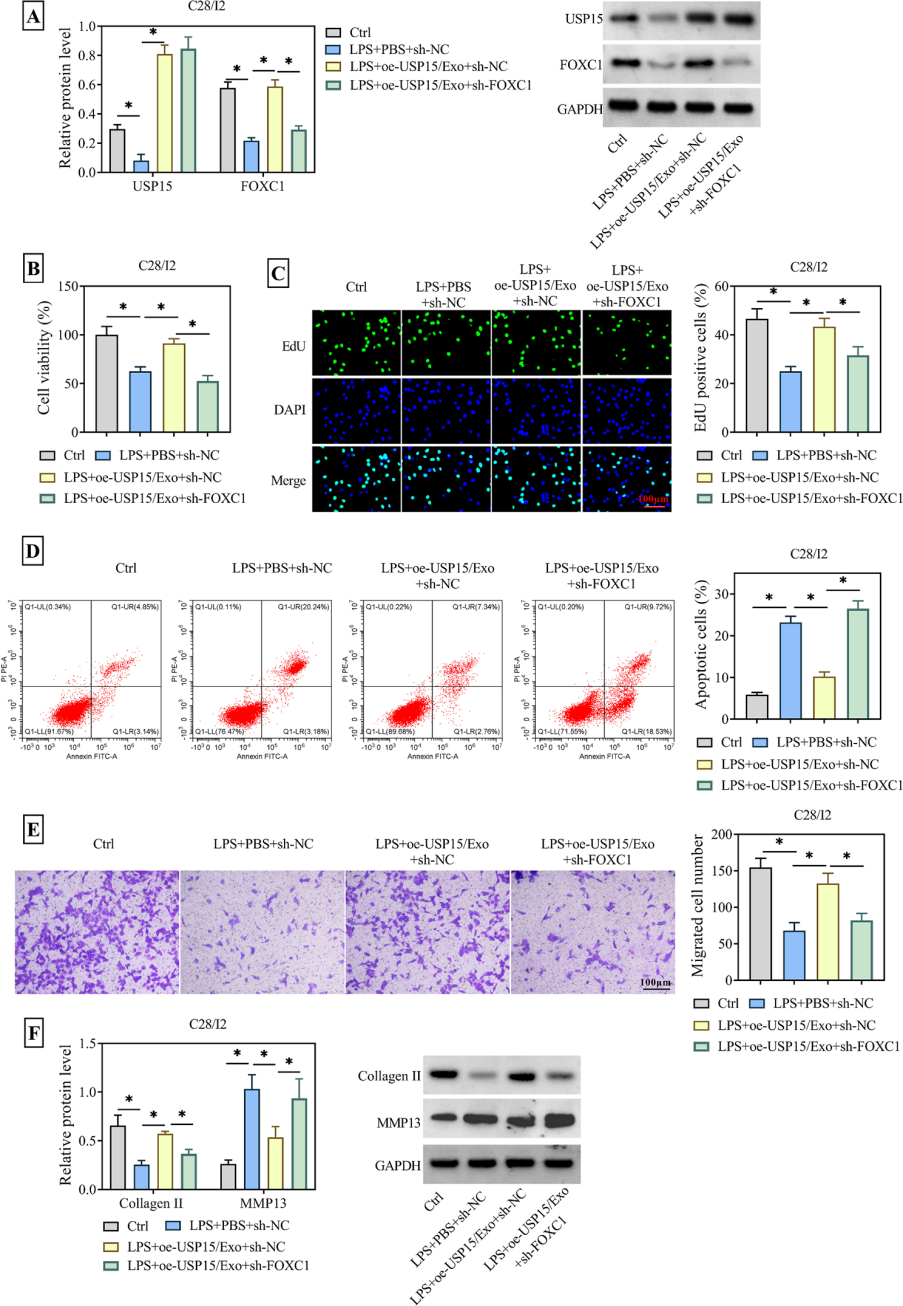
Silencing of FOXC1 restores the effects of USP15-modified Exos on LPS-injured chondrocytes. C28/I2 cells were randomly divided into four groups: Ctrl group; LPS + PBS + sh-NC group; LPS + oe-NC/Exo + sh-NC group; LPS + oe-USP15/Exo + sh-FOXC1 group. (**A**) WB assay was used to determine the protein levels of USP15 and FOXC1 in C28/I2 cells stably transfected with sh-FOXC1 after co-incubation with oe-USP15/Exo. (**B**) C28/I2 cells viability was measured by CCK-8 kit. (**C**) EdU assay was used to measure C28/I2 cells proliferation. (**D**) The apoptosis rate was analyzed by flow cytometry. (**E**) Transwell assay was used to observe C28/I2 cells migration behavior. (**F**) The protein expression of Collagen II and MMP13 were measured by WB assay. *P < 0.05

Based on the above results, it was hypothesized that USP15 and FOXC1 might play a role in chondrocyte injury. LPS-treated C28/I2 cells were treated with Exos overexpressing USP15 or additionally transfected with sh-FOXC1. WB results showed that FOXC1 protein level was greatly downregulated after transfection with sh-FOXC1 (Fig. 6A). Subsequent assays using CCK-8 and EdU kits displayed that the enhanced cell viability and proliferative behavior due to oe-USP15/Exo were both decreased when FOXC1 was silenced (Fig. 6B-C). In contrast, the apoptosis rate of C28/I2 cells, which was effectively decreased due to co-incubation with oe-USP15/Exo, re-increased after transfection with sh-FOXC1 (Fig. 6D). At the same time, as shown in Fig. 6E, the migratory behavior of cells was hindered after LPS treatment, whereas oe-USP/Exo could restore its migratory ability, but it was destroyed again by the presence of sh-FOXC1. Besides, the expression of Collagen II was inhibited but MMP13 was upregulated in the LPS + oe-USP15/Exo + sh-NC group compared with the LPS + PBS + sh-NC group. However, after transfection of sh-FOXC1 into C28/I2 cells, the originally high level of Collagen II was suppressed, while the originally low expression MMP13 was re-upregulated (Fig. 6F). That is to say, the Exos of USP15 overexpressed achieved the repair effect on LPS-induced chondrocyte damage by expediting the level of FOXC1.

## Discussion

MSC-derived Exo therapy is a cutting-edge biological treatment method, which is based on the biological characteristics of Exos secreted by MSCs and plays an important role in the treatment of many diseases [20, 21]. Although stem cell Exo therapy has demonstrated enormous application potential, natural Exos face issues such as product heterogeneity, rapid elimination in the body, and poor long-term stability during preservation. Therefore, in order to maximize the therapeutic effect of Exos, it is necessary to conduct genetic modification on them. A study indicated that Exos derived from human umbilical cord MSCs modified with aHSCs targeting peptides had a significant therapeutic effect on pulmonary fibrosis [22]. Another report has confirmed that MSC-derived Exos overexpressing HIF-1α could promote angiogenesis and thereby mediate cardioprotective effects in patients with myocardial infarction [23]. It is noteworthy that multiple studies have confirmed the ability of ADMSC to induce cartilage regeneration in patients with OA. Additionally, ADMSC can secrete VEGF and TGF-β. These growth factors effectively stimulate the proliferation and differentiation of surrounding cells, further promoting the formation of new blood vessels and improving local blood supply. This series of biological effects not only helps to alleviate pain in patients but also significantly enhances joint function [24–26]. Meanwhile, evidence has shown that the deubiquitinating enzyme USP15 is also involved in cartilage repair and the regulation of OA progression [18]. In light of these findings, we cannot help but ponder: Could the modification of Exos derived from ADMSC with the USP15 gene be an effective means to alleviate the symptoms of OA? Firstly, we utilized Oil Red O and Alcian Blue staining techniques to verify the multidirectional differentiation potential of cells isolated from adipose tissue, which have the ability to differentiate into adipocytes and chondrocytes. Additionally, flow cytometry analysis confirmed the expression of specific ADMSC markers CD90 and CD44 on these cells. Subsequently, the pcDNA3.1-USP15 vector was introduced into well-grown ADMSC cells to overexpress the USP15 gene, and Exos were successfully isolated using magnetic bead separation technology. With the aid of transmission electron microscopy and NTA techniques, we observed that the average vesicle diameter of these Exo particles was approximately 100 nm. The WB and RT-qPCR results all confirmed the successful isolation of Exos overexpressing USP15 from ADMSC.

Macrophages, as an important component of the human immune system, are subdivided into M1 and M2 types based on their functional characteristics and surface markers. Among them, M1 macrophages dominate the inflammatory response and exacerbate tissue damage; in contrast, M2 macrophages exhibit anti-inflammatory properties and contribute to tissue repair and regeneration [27]. Numerous studies have indicated that Exos secreted by MSCs can effectively promote the transition of macrophages from the M1 type to the M2 type and enhance the release of anti-inflammatory factors and chemokines [28]. Therefore, inducing macrophage polarization to M2 type is expected to become a new strategy for the treatment of OA. Fang et al. have demonstrated that TREM2 induced M2 polarization of macrophages by regulating the NF-κB/CXCL3 axis, thereby effectively alleviating OA [29]. Besides, another study found that BMP-7-modified synovial MSC-derived exosomes could also induce M2 polarization of macrophages and improve the symptoms of OA [30]. Thus, we hypothesized that Exos secreted by ADMSCs overexpressing USP15 may also have an impact on macrophage polarization and chondrocyte damage in OA. After co-culturing DiI-labeled oe-USP15/Exo with THP1-M0 and C28/I2 cells, observation under a fluorescence microscope confirmed that the Exos had been successfully internalized by cells. And the high expression of USP15 was confirmed using both WB and RT-qPCR techniques. The THP1-M0 and C28/I2 were treated with LPS in order to establish an OA symptom model. Through the analysis of flow cytometry and ELISA assays, it was revealed that the presence of oe-USP15/Exo induced the polarization of THP1-M0 cells towards M2 and stimulated the release of anti-inflammatory factors. Simultaneously, Exos overexpressing USP15 repaired the damage caused by LPS to chondrocyte viability, proliferation, and migration, and effectively reduced the apoptosis rate. Meanwhile, WB analysis showed that oe-USP15/Exo upregulated the level of Collagen II, a collagen protein involved in chondrocyte remodeling, while blocking the levels of proteins related to extracellular matrix degradation.

To delve deeper into the mechanism of USP15-modified Exos in the treatment of OA, we utilized the Ubibrowse_v2 website to predict the top 20 target proteins of USP15 and screened out the FOXC1 protein with the highest score. FOXC1 is a transcription factor belonging to the “C” subfamily of the FOX/Winged helix family, and it participates in various biological processes such as cell proliferation, differentiation, apoptosis, and organ development. A large number of clinical studies have shown that increased FOXC1 expression is closely related to poor prognosis in many cancer subtypes [31, 32]. Interestingly, Yuan et al. suggested that microRNA-138-5p was involved in the IL-1β-induced cartilage degradation process in human chondrocytes by targeting FOXC1 [33]. So will USP15-modified Exos exert a therapeutic effect on OA through FOXC1? It was observed that the level of FOXC1 was increased after USP15 was overexpressed in THP1-M0 and C28/I2 cells. And then, the interaction between USP15 and FOXC1 was further determined by GST pull-down and Co-IP techniques. Additionally, after treating with CHX, we found that overexpressing USP15 could mitigate the effect of CHX on FOXC1, suggesting that USP15 had a role in stabilizing the level of FOXC1 within the THP1-M0 and C28/I2 cells. And precisely because USP15 was a deubiquitinating enzyme, it was determined that through exogenous ubiquitination experiments that overexpressing USP15 reduced the ubiquitination level of FOXC1. In other words, USP15 stabilized the intracellular level of FOXC1 by targeting it and exerting its deubiquitinating function. Subsequently, we repeated the aforementioned experiments and implicated that, after FOXC1 was silenced, THP1-M0 cells repolarized towards the M1 phenotype and the release of proinflammatory cytokines in the cell supernatant notably increased. Additionally, the silencing of FOXC1 ameliorated the therapeutic effect of oe-USP15/Exo on LPS-injured chondrocytes.

In summary, our research findings elucidates that USP15-modified ADMSCs-derived Exos induces macrophage polarization towards the M2 phenotype by promoting FOXC1 deubiquitination, thereby effectively reducing the degree of chondrocyte damage in OA. Unfortunately, this study is mainly conducted in cellular and has not yet been validated in animal models and clinical patients. Therefore, further validation of these findings in clinical studies is needed in the future.

## Data Availability

No datasets were generated or analysed during the current study.
